# Wheat Ear Segmentation Based on a Multisensor System and Superpixel Classification

**DOI:** 10.34133/2022/9841985

**Published:** 2022-01-28

**Authors:** Alexis Carlier, Sébastien Dandrifosse, Benjamin Dumont, Benoît Mercatoris

**Affiliations:** ^1^Biosystems Dynamics and Exchanges, TERRA Teaching and Research Centre, Gembloux Agro-Bio Tech, University of Liège, 5030 Gembloux, Belgium; ^2^Plant Sciences, TERRA Teaching and Research Centre, Gembloux Agro-Bio Tech, University of Liège, 5030 Gembloux, Belgium

## Abstract

The automatic segmentation of ears in wheat canopy images is an important step to measure ear density or extract relevant plant traits separately for the different organs. Recent deep learning algorithms appear as promising tools to accurately detect ears in a wide diversity of conditions. However, they remain complicated to implement and necessitate a huge training database. This paper is aimed at proposing an easy and quick to train and robust alternative to segment wheat ears from heading to maturity growth stage. The tested method was based on superpixel classification exploiting features from RGB and multispectral cameras. Three classifiers were trained with wheat images acquired from heading to maturity on two cultivars at different levels of fertilizer. The best classifier, the support vector machine (SVM), yielded satisfactory segmentation and reached 94% accuracy. However, the segmentation at the pixel level could not be assessed only by the superpixel classification accuracy. For this reason, a second assessment method was proposed to consider the entire process. A simple graphical tool was developed to annotate pixels. The strategy was to annotate a few pixels per image to be able to quickly annotate the entire image set, and thus account for very diverse conditions. Results showed a lesser segmentation score (F1-score) for the heading and flowering stages and for the zero nitrogen input object. The methodology appeared appropriate for further work on the growth dynamics of the different wheat organs and in the frame of other segmentation challenges.

## 1. Introduction

Grain yield is the most valuable trait for the wheat breeders, as it clearly translates the economic value of the selection process. However, breeders are interested in improving the selection pipelines with other crop performance criteria such as the radiation use efficiency [1]. Recent advances in sensing technologies and the increase of computing power nowadays allow to extract lots of information from a crop canopy in a nondestructive way and throughout all the season [2]. In particular, cameras were mounted on terrestrial vehicles, or low altitude UAV have the capacity to acquire high-resolution images in the field where the different organs of wheat can be distinguished. Multisensor systems allow to use different sources of data to enrich each data point. [3] used five sensing modules in a soybean plant breeding program. They reported strong correlation among sensor-based plant traits providing a powerful tool for phenotype characterization.

In a canopy, leaves, tillers, and ears respond to different development patterns and roles. Therefore, the high-throughput phenotyping approaches should consider their respective contributions to the sensor data. This is particularly true from the emergence of the ears. [4, 5], and [6] reported an effect of the presence of the ears in the canopy impacting image signal at UAV scale. [7] pointed out the lack of work for the postflowering period when ears are intercepting more and more light. Besides, ear number is strongly correlated to final grain yield and some wheat diseases, like Fusarium head blight, arise only on the reproductive organs. As a result, the automatic segmentation of ears in the images appears as a critical part of the computer vision process. This task however remains challenging because of the overlaps between the organs and the multiple color and architecture variations induced by different development stages, cultivars, and light conditions.

Deep learning methods, based on convolutional neural network (CNN), are currently the state-of-the-art computer vision tools for plant phenotyping [8]. They reach good performances for ear counting tasks using RGB images [9–11], but they often stick to the detection of bounding boxes around the ears, without providing the segmentation mask. [12] achieved a satisfactory segmentation on images of wheat at flowering stage despite a computation time of 18 seconds per image. Yet, CNNs have disadvantages: (i) they require a lot of training data which demands time and money, (ii) the different underneath processes are not yet totally understood [8], and (iii) they remain complicated to configure and process. To overcome these inconveniences, alternative approaches have been proposed for ear detection and segmentation. [13] tested thermal imagery for ear counting, but the method was not robust enough. [10] achieved good counting results with a mixed model based on superpixels and deep neural networks. [14, 15], and [16] used machine vision based on textural features with good segmentation in diffuse light conditions. [17] explored the recognition of wheat ears from multisensor data computed with hand-crafted machine vision techniques. They achieved good results only for well illuminated images. [18] used a SVM classifier of superpixels for ear segmentation on a single date. They noticed an impact of fertilization on the classification accuracy. [19] estimated a postharvest ear density, based on residual stems, with a relative RMSE close to 7%. Nevertheless, most of the previous studies used few dates of acquisition, and the segmentation performances were rarely assessed.

The objective of this paper is to propose a simple method for automatic wheat ear segmentation. The method relies on the classification of simple linear iterative clustering (SLIC) superpixels using features extracted from the fusion of RGB and multispectral images. The accuracy of the superpixel classification has been evaluated. Moreover, the entire process of segmentation has been assessed by means of an independent method. The segmentation performances were analyzed and discussed for two winter wheat cultivars with different levels of fertilization, imaged from heading to maturity development stages.

## 2. Materials and Methods

### 2.1. Data Acquisition

#### 2.1.1. Material

**Figure 1:**
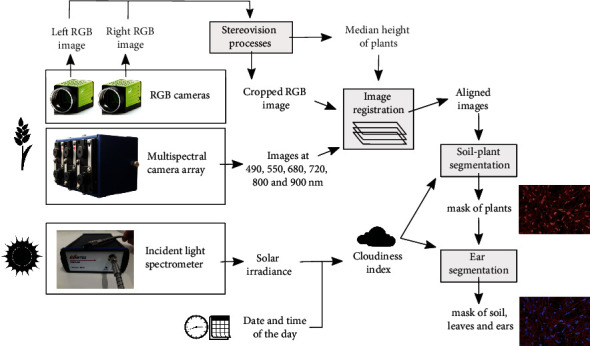
Image processing pipeline from field images to ternary mask.

A sensor pod combining multispectral and 3D vision was used in this study. The multispectral camera array was a Micro-MCA (Tetracam Inc., Gainesville, FL, USA) (Figure 1). It consisted of six monochrome cameras equipped with 1280 × 1024 pixels of CMOS sensors. The optical filters were narrow band-pass filters centered at 490, 550, 680, 720, 800, and 900 nm. The width of each band was 10 nm except for the 900 nm band that had a width of 20 nm. The lenses had a focal length of 9.6 mm and an aperture of f/3.2. Additionally, two RGB cameras were used to record color and 3D information by stereovision. Those devices were GO-5000C-USB cameras (JAI A/S, Copenhagen, Denmark). The distance between the centers of the two sensors was 50 mm. Each camera was equipped with 2560 × 2048 pixels of CMOS sensor and a LM16HC objective (Kowa GmbH, Düsseldorf, Germany). Their focal length was 16 mm, and the aperture was set to f/4.0. The incident light sensor (ILS) associated to the sensor pod was a AvaSpec-ULS2048 equipped with a cosine corrector (Avantes, Apeldoorn, Netherlands). The irradiance calibration was carried out in the factory on March 23, 2020, just before the measurement campaign.

#### 2.1.2. In-Field Data Acquisition

Images and incident light spectrums were acquired during the 2020 season in two field trials located in the Hesbaye area, Belgium (50° 32′ 40^″^ N and 4° 44′ 56^″^ E), on homogenous deep silt loamy soil and a temperate climate. The first trial (trial 1) was planted with winter wheat cultivar “Mentor” on November 7th, 2019. The second trial (trial 2) was planted with winter wheat cultivar “LG Vertikal” on November 5th, 2019. Both trials were sowed with a density of 250 grains/m^2^. The experimental microplots measured 1.95 × 6 m^2^, and the row spacing was 0.14 m. The microplots were fertilized three times (at BBCH stages 28, 30, and 39) with 27% ammonium nitrate. Trial 1 consisted of a complete randomization of eight objects of contrasted nitrogen inputs repeated in four blocks. Trial 2 consisted of a complete randomization of sixteen objects combining contrasted nitrogen inputs and fungicide applications (0, 1, 2, or 3 dates of fungicide treatment) repeated in four blocks. Images were acquired from heading stage to maturity stage on 9 dates for trial 1 (June 3rd, June 11th, June 18th, June 23rd, June 29th, July 7th, July 13th, July 22nd, and July 29th) and on 7 dates for trial 2 (June 2nd, June 9th, June 16th, June 26th, July 7th, July 13th, and July 22nd). At each date and for each camera, four images were taken per microplot.

A phenotyping platform was designed to capture nadir frames of wheat microplots (Figure S1). The sensor pod was installed on a cantilever beam to avoid shadows from the rest of the platform in the images. The height of that pod was adjusted at each acquisition date to keep a distance around 1.6 m between the cameras and the top of the canopy. At this distance, the footprint of the frames was 0.98 m^2^ for the cameras of the multispectral array and 1.26 m^2^ for the RGB cameras. The images were recorded using a color depth of 10 or 12 bits per pixel then reduced to 8 bits per pixel, because the stereovision and registration open-source libraries need 8-bit inputs. The autoexposure algorithms of the RGB and multispectral devices were adapted to prevent image saturation as suggested by [20]. The ILS was positioned above the cameras. A spectrum of the incident sunlight was recorded at each image acquisition using a 16-bit resolution. Each recorded spectrum was the average of three consecutive measurements. It was corrected for dark noise and nonlinearity of pixel response to exposure time. Thanks to the factory calibration, digital values were converted to irradiance data. Each acquisition of images and their associated solar spectrum took only a few seconds. It corresponded to the time necessary to average the spectrums and ensure a proper exposure time for all the cameras.

### 2.2. Image Preprocessing

#### 2.2.1. Image Registration

The images from the multispectral camera array and the RGB images were registered using a B-spline-based method [21, 22] (Figure 1). After this operation, the images could be aligned pixel to pixel to form a single multichannel image containing the multispectral and RGB information at the pixel level. In order to diminish the impact of potential registration errors, a blur filter was applied on each channel.

#### 2.2.2. Soil-Plant Segmentation

**Figure 2:**
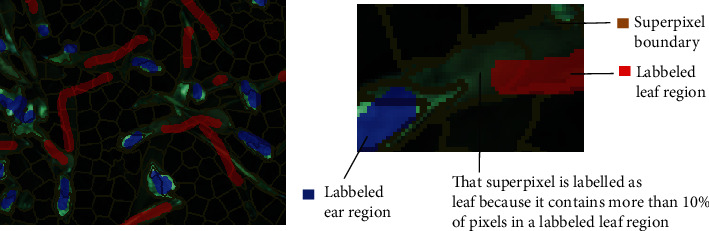
Illustration of the superpixel labelling process.

**Figure 3:**
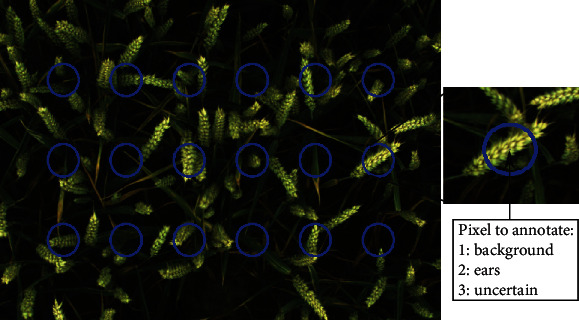
Illustration of the pixel-based evaluation.

**Figure 4:**
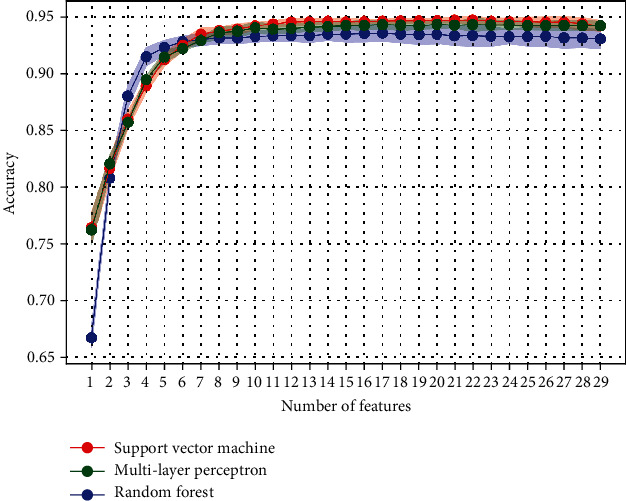
Sequential backward feature selection for the three classifiers. The transparent color areas refer to the standard deviation of the accuracy from the cross-validation.

**Figure 5:**
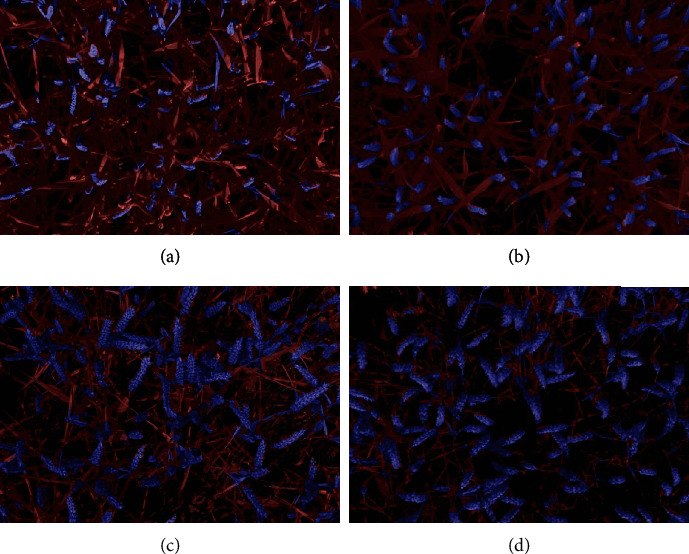
Wheat canopy segmentation at the organ scale. The soil segmented from 800 nm band appears in dark grey, the leaves in red, and the ears in blue. The segmentation is illustrated for the following growth stages: (a) beginning of flowering (6 DAH), (b) medium milk (21 DAH), (c) early dough (46 DAH), and (d) maturity (62 DAH).

The separation between the soil and all the plant elements was based on a thresholding approach applied on the 800 nm channel (red mask in Figures 1–5). The threshold value was automatically determined for each image based on the first local minimum of the histogram. That simple method could be used because of the significant reflectance difference of the plant and the soil in the NIR. However, for some cases where strong direct sunlight reached the soil, a few soil pixels could be confused with the low and shaded leaves. To avoid that confusion, a threshold in the blue channel was added for the pixels of low NIR values. The need to add this second step was judged thanks to a cloudiness index derived from the ILS measurement at the time of image capture. A threshold of 0.90 was empirically determined. The cloudiness index was built as follows:

(1) $$\begin{array}{c} C_{T}=1-\frac{E}{E_{0}*\cos\left(z\right)}, \end{array}$$

where *E* is the solar irradiance (W/m^2^) in the spectral measurement range of the spectrometer, *E*_0_ is the solar constant (1360 W/m^2^), and *z* is the sun zenith angle.

### 2.3. Plant-Ear Segmentation

#### 2.3.1. Superpixel Classification and Evaluation

The most challenging canopy segmentation step was the separation of the plant elements into ears and leaves, and the latter may contain the few visible stems' parts. The segmentation was based on superpixels, i.e., groups of pixels sharing common characteristics such as color and spatial information. The superpixels were computed on RGB images, and the resulting regions were extended to the other channels. The superpixel algorithm was the simple linear iterative clustering (SLIC) that has already been used in the case of ear segmentation by [10, 12, 23], and [18]. Such a method is based on k-means clustering using five features from CIELAB color space and pixel coordinates. This was implemented by means of the Python 3 scikit-image library (version 0.17.2.). Three main parameters were optimized to suit all development stages of the crop. They were the approximate number of superpixels in the output, the compactness, and the maximum number of iterations of k-means. Their values were set to 1500, 10, and 30, respectively. This set of parameters gave the best classification results as it allowed a good trade-off between too big or too small superpixels.

Three machine learning algorithms were tested: random forest (RF), multilayer perceptron (MLP), and support vector machine (SVM). They were chosen for their ability to perform segmentation tasks and for their capacity to deal with correlated features. The accuracy score was used as metric to evaluate the different models and for the training phase. The accuracy was defined as follows:

(2) $$\begin{array}{c} \text{Accuracy}=\frac{tp+tn}{tp+tn+fp+f\text{n}}, \end{array}$$

where *tp*, *tn*, *fp*, and *fn*, respectively, stand for the number of true positives, true negatives, false positives, and false negatives from the confusion matrix.

The algorithms were fed with the following features: the average value of the superpixels for the six monochrome channels, the normalized RGB channels, the hue-saturation-value (HSV) channels, and fifteen vegetation indices (Table S1). Two features unrelated to image content were added: the number of days after sowing (DAS) and the cloudiness index. They were added to take into account the growth stage of the wheat and the lighting conditions, respectively.

Prior to the classification step, these features were scaled using a standard scaler, i.e., removing the mean and scaling by the standard deviation. The hyperparameters were then optimized on the 29 features. A set of values were tested for the different hyperparameters of each classifier. The hyperparameters for the SVM were the kernel type, the C, and the gamma. Those of the MLP were the hidden layer size, the activation function, and the alpha. Those of the RF were the number of estimators, the maximum depth, and the minimum number of samples required to be at a leaf node or to split an internal node. A sequential backward feature selection was then performed to avoid eventual noises from redundant features and to extract relevant features allowing the classification. At each step, the algorithm generated all possible feature subsets of size *n* − 1 (*n* stands for the total number of features) and evaluated each subset using a 5-fold cross-validation. The feature to remove was the feature absent from the subset with the highest accuracy. Features were removed one by one, until reaching the desired minimum of features. Finally, a second step of hyperparameter optimization was done with the selected features. Each step of the pipeline was evaluated in a 5-fold cross-validation on the training set.

#### 2.3.2. Annotation and Training Dataset

The superpixels were labelled into leaves and ears to create training and test datasets for supervised machine learning. The labelling was made on RGB images using an online machine learning platform [24]. A particular attention was paid to select images representative of the diversity of all the acquisitions, that is, nitrogen input, growth stages, and lighting conditions. At least two images from each date were chosen for labelling, resulting in a dataset of 43 images. Labelled regions were drawn by hand by dragging a brush-like pointer (Figure 2). Care was taken to consider the diversity of leaves and ears and to select approximatively the same amount of data for the two classes (46% labelled as ears). The labelled regions were then converted to labelled superpixels. Those which included at least 10% of labelled pixels belonging to a class were considered of this class. The superpixel containing labelled pixels for both classes or containing less than two pixels was discarded. A total of 15,765 superpixels were selected, randomized, and split into 80% training and 20% test.

### 2.4. Pixel-Based Segmentation Evaluation

The evaluation of the segmentation method at the pixel level could not be limited to the evaluation of the superpixel classification. A supplementary evaluation was needed to consider the entire process of segmentation. A protocol was designed to perform a quick and easy evaluation allowing a first comparison of the performances for the different dates and fertilization objects. To reach this objective, a custom annotation tool was created. The method consisted in annotating 18 pixels distributed in three rows (Figure 3) on each RGB image. That configuration was judged a good trade-off between representing the image heterogeneity and having a quick tool to process a maximum number of images. The tool automatically zoomed on each pixel. Then, the operator pushed one of three buttons to attribute a class to this pixel: class 1 for the background (soil, leaves, stems,…), class 2 for the ears, and class 3 if it was not possible to decide between class 1 or 2. It happened that the operator could not distinguish well what the pixel represented or that pixel was located at the edge between an ear and the background. That procedure was executed for all the images for half of the dates from heading to maturity. The maturity occurred around 62 days after heading (DAH).

To statistically compare those human annotations with the predicted segmentation, the F1-score was used [11, 12]. It is particularly adapted for unbalanced classes, as the ones in this problem.

(3) $$\begin{array}{c} \text{F}1‐\text{score}=2\times \frac{\text{precision}\times \text{recall}}{\text{precision}+\text{recall}},\\ \text{Precision}=\frac{tp}{tp+fp},\\ \text{Recall}=\frac{tp}{tp+fn}. \end{array}$$

Finally, annotations from three different operators were compared to estimate the possible bias resulting from this quick human annotation. The metric used for this comparison was the Cohen's kappa. It is known to be more robust than the accuracy parameter since it accounts for the probability of true values occurring randomly:

(4) $$\begin{array}{c} \kappa =\frac{p_{o}-p_{e}}{1-p_{e}}, \end{array}$$

where *p*_*o*_ is the empirical probability of agreement on the label assigned to any sample (the observed agreement ratio) and *p*_*e*_ is the expected agreement when both operators assign labels randomly, according to scikit-learn documentation. Labels of class 3 were discarded as they referred to uncertain annotations.

## 3. Results

### 3.1. Superpixel Classification

#### 3.1.1. Feature Selection

Figure 4 presents the accuracy of the three classifiers in a 5-fold cross-validation according to the number of features. Classifier hyperparameters used in this process were firstly tuned with all the features. Then, a feature selection process was performed, aiming at establishing a set of features such as it reaches the maximum accuracy (when the curve reaches a plateau) with a minimum number of features.  Table 1 shows the selected feature sets that were manually selected according to the two previous conditions. Looking down to the one-feature models, it appears that the selection process applied on the three classifiers yielded the same feature: the value component from the HSV of the color image.

#### 3.1.2. Classifier Accuracy

The overall accuracy of superpixel classification for RF, MLP, and SVM algorithms on the test set was, respectively, 0.93, 0.94, and 0.94. Regarding the important heterogeneity of the dataset, these results are considered good. Even in difficult scenes, strongly impacted by sunlight, they provided acceptable segmentation as illustrated in Figures 5(a) and 5(c). SVM was chosen over MLP because of its simplicity to use and to tune. The learning curve of the SVM and the MLP showed a convergence between training and test sets, indicating that the model was not underfitting or overfitting (Figure S2), whereas the RF showed an overfitting regarding the training curve staying close to an accuracy of 1. After tuning, the C regularization parameter was set to 100, the chosen kernel was a radial basis function, and the kernel gamma coefficient was set to 0.1.

### 3.2. Pixel-Based Evaluation

#### 3.2.1. Human Annotation Analysis

Human annotation has rarely been evaluated in previous studies, yet it is also a source of error in the calculation of the final metric. A class “uncertain” was added to the annotation tool to build the cleanest dataset possible in a quick way. This label was attributed to 3.2% of the total amount of pixels, ranging from 1.3 to 4.5% depending on the considered date. It mainly concerned pixels at the edges between the ears and the background or pixels difficult to identify, for example, in shaded zones. Depending on nitrogen object and date, 62 to 77% of the pixels labelled “uncertain” have been predicted as background.

Three annotations from different human operators were compared on the best date, i.e., on June 18th. Cohen's kappa coefficients of 0.79, 0.75, and 0.78 between the different operators were observed, which can be interpreted as “good to strong” agreement and thus validate the annotation methodology used.

#### 3.2.2. Impact of Growth Stage and Nitrogen

**Figure 6:**
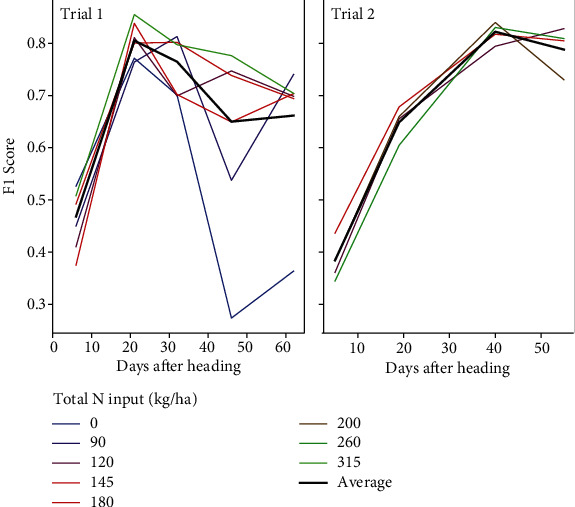
Temporal curve of the F1-score for both trials according to the total nitrogen input.

In average, sets of 3345 and 4460 reference pixels, respectively, for trials 1 and 2 were used to calculate a F1-score for each date. They represented the total amount of pixels annotated reduced by the number of label “uncertain,” i.e., 3.2% in average. The overall F1-score was 0.72 with a precision of 0.70 and a recall of 0.74. It gave acceptable segmentation as illustrated in Figure 5. The periods of heading and flowering seem to be the worst moments for ear segmentation (Figure 6). At these periods, ears are still growing, and their color is very similar to the leaf color. The best scores were obtained from 10 to 15 DAH until the beginning of ripening around 40 DAH. F1-scores above 0.80 yielded a very good segmentation illustrated in Figure 5(b). Those scores were obtained on cloudy days. The absence of direct sunlight resulted in homogeneous light conditions and in the absence of shadows.

The zero nitrogen object shows a bad F1-score from the moment the first signs of senescence appeared, i.e., around 30 to 40 DAH.

#### 3.2.3. Ear Ratio

**Figure 7:**
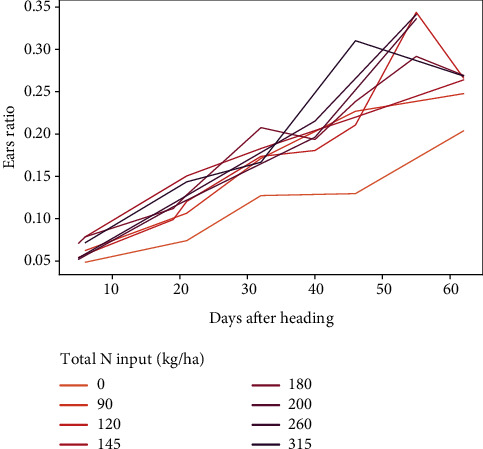
Temporal curve of ear ratio according to nitrogen input for both trials.

One relevant information arising from the pixel-based annotation is the proportion of each label.  Figure 7 presents the dynamics of the ear proportion, called ear ratio, according to the nitrogen objects for both trials. The observed values between 5 to 35%, in accordance with [25], represent the proportion of pixels noted as ears in the images. The curves can be seen as the physiological growth of the wheat ears. We can notice that the zero nitrogen object differed from the others as the season advanced. That can be explained by the fact that the density of ears is strongly related to nitrogen input; thus, less ear pixels were present in the image.

## 4. Discussion

The proposed segmentation of a wheat canopy into leaves and ears was performed in two steps: the creation of superpixels and their classification. Image analysis techniques were used to extract relevant features in order to classify the superpixels into ear and the rest of the plant. Nevertheless, it remains interesting to proceed to a feature selection to distinguish the most relevant features. The sequential backward feature selection used on the three classifiers allowed to extract feature sets to achieve the best accuracy with a minimum number of features. Even in a context of medium to strong collinearity (Figure S3), classifiers somewhat agreed on the selected features that strengthened their significance in the classification task; the common features were cloudiness index, DAS, hue, saturation, value, B, and mNDblue. Others selected features were related to 800 nm, 680 nm, and 720 nm bands. [4] also observed high relative reflectance differences between canopy with and without ears, up to 85% for the red band and up to 34% for the NIR band. The DAS feature was introduced to account for the growth stage of the crop. Depending on the air temperatures all along the season, a same growth stage could however be reached at a different number of days after sowing. To better account for the growth stages and be able to use the model for several years, it would be advised to use growing degree days instead of DAS. The objective to introduce the vegetation indices and the cloudiness index was to deal with the illumination heterogeneity encountered. It appeared that they were also part of the selected features. Looking down to the one-feature models, the three classifiers have picked out the same feature: the value feature from the HSV color space. Wheat ears usually appeared brighter and can be segmented using a simple thresholding method. That single feature already gives acceptable accuracy (Figure 4). [26] reached also very good results using the Otsu method. Nevertheless, multifeature method had improved their results. Concerning our study, the best accuracy was reached with features from all the sensors. Minor accuracy differences were observed between the three classifiers on the test set. Thus, it can be acknowledged that the selected features were well describing the differences between the ear and the rest of the plants, and that the multisensor approach was relevant for this segmentation challenge. Other methods based on predefined pixel features such as color or textural parameters were tested and worked well with RGB images in diffuse light conditions. However, strong direct light conditions were not suitable at all [17, 27].

**Figure 8:**
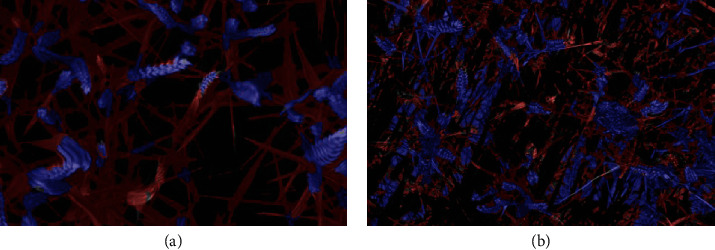
Illustrations of preprocessing imperfections: (a) weird deformations created by the registration process and (b) soil segmentation error.

The proposed segmentation is the result of multiple processes from image acquisition to final ear mask. Thus, it remains hard to identify whether errors came from the classification or from the preprocesses. That is why we proposed an evaluation method based on the quick annotation of single pixels considered as ground truth. This method permitted to assess the whole process of segmentation comprising image registration, soil segmentation, superpixel construction, and their classification. Consequently, it is not very pertinent to compare the F1-score obtained in this study, based on a few pixels from all the images, with F1-scores obtained in other studies, based on other evaluation methodologies. However, the good to strong agreement between the three annotators suggests a good stability of the proposed evaluation method. Both trials indicate lower F1-score from heading to the end of flowering. At these growth stages, leaves and ears have very similar colors; thus, the classification based on color features is more complicated. Lesser F1-score observed for the zero nitrogen object was also reported by [18]. Our hypothesis is that the physiology and the growth dynamics of this object are really different than the others. It includes an earlier senescence, some nitrogen stress symptoms (light green leaves), and a lower leaf area index. Other sources of errors have been identified. Firstly, the soil segmentation was not exempt from errors. This could especially be the case in well-illuminated scenes where the strategy to combine 800 nm and 490 nm bands was not perfect (Figure 8(b)). This phenomenon could be amplified in the case of zero nitrogen objects where the soil was more present in the images. Secondly, image preprocessing could also lead to segmentation errors; raw images were in a first step prompted to image registration. [21] mentioned some imperfections during this preprocessing. Even though an erosion operation and a blur filter were applied, weird organ deformations were sometimes observed in windy situations (Figure 8(a)).

The SLIC superpixel algorithm was parametrized to generate regions that were smaller than the organs. A leaf or an ear was generally described by several superpixels. Each superpixel gathered similar and spatially close pixels, and its edges were often well delimited. Nevertheless, some superpixels integrated both leaf and ear parts and were responsible for segmentation errors. It particularly occurred in strong illumination scenes. Such a phenomenon was also noticed by [10]. It is a disadvantage compared to a classification method at the pixel level. However, the noise created by elements misclassified at the pixel level may be integrated in a superpixel of the correct class, which makes the segmentation clearer. The superpixel approach is also very useful to build the training and test datasets. During the study, it was observed that adding only two images from a new date to the training dataset was sufficient to reach a good segmentation of the concerning date. Thanks to the method illustrated in Figure 1, it was possible to label pixels that were not selected by hand, because they lied in the same superpixel as the selected elements. Furthermore, features of a superpixel represent the average of all the pixels of this zone. That prevents some outlier pixels and substantially decreases the size of the final database. To avoid attributing wrong labels, the operator should pay attention to not tracing training label zones in superpixels containing both leaf and ear elements. 4% of the labelled superpixels were discarded before classification because they contained labels from both classes. That selection method was faster than traditional pixel selection. It allowed to include pixels that would have been difficult to select visually (pixels on the edges of the organs). It is also quicker while maintaining an acceptable segmentation than recent CNN models. For instance, [28] needed to reexamined and complemented their original Global Wheat Head Detection dataset. They reach up to 4700 RGB images which has demanded a huge amount of annotating time. Moreover, CNN remains hard to implement and demands a sufficient computing resource. Finally, segmentation is often evaluated on handmade reference masks that are very time-consuming and thus limited to a few images. The proposed evaluation method based on the quick annotation of single pixels allowed to annotate a large number of images and thus observe the trends between different dates or fertilization objects.

The whole proposed methodology, comprising the superpixel technique and the evaluation method, can also be used in the frame of other types of segmentation challenges. [29] used the SLIC algorithm with features extracted from CNN to detect invasive plants. It can also be used to elaborate a CNN dataset of wheat ear patches considering only the high probability patches from a classifier.

## 5. Conclusions

An automatic segmentation of wheat ears based on superpixel classification was proposed. Features from RGB and multispectral cameras were fed to three classifiers. A training database of two trials with different levels of nitrogen from heading to maturity was set. The annotation process was made easier and faster thanks to the superpixels created by the SLIC algorithm. The tuned SVM classifier reached 94% accuracy but was not sufficient to assess the overall segmentation. In order to validate the whole pipeline, a pixel-based evaluation method was proposed. It consisted of quick annotation of single pixels for all the images per date. The results showed an overall F1-score of 0.72 yielding good segmentation. Lower score was observed around anthesis date for both trials and for the zero nitrogen input object. The fusion of images from multiple cameras brought a real added value to the segmentation task. Features from RGB, multispectral cameras, and incident light sensor were selected by the three classifiers. The proposed segmentation and evaluation methods were fast and easy ways to accurately segment wheat ears with a small to medium size database. It seems a good compromise between methods based on predefined image features and recent deep learning methods. The method can be used for other purposes than ear segmentation with special attention to the training set. It is also a great avenue for further studies on the development of wheat organ reflectance from heading to maturity.

## Figures and Tables

**Table 1:** Selected features.

Classifier	Features	Number
SVM	Cloudiness index, DAS hue, saturation, value, G, B, 680 nm, SR, NDVI, NDRE, VARI, mNDblue	13
MLP	Cloudiness index, DAS, hue, saturation, value, G, B, 900 nm, GNDVI, RDVI, OSAVI, NDRE, TCARI, CIrede, mNDblue	15
RF	Cloudiness index, DAS, hue, saturation, value, B, NDVI, mNDblue	8

## Data Availability

Data and the annotation tool mentioned in this study are available on request from the corresponding author.

## Supplementary Materials

Supplementary Materials Table S1: description of the 15 vegetation indices and the normalized RGB components used as features for the machine learning algorithms aiming at separating leaves and ears. Figure S1: phenotyping platform with the sensor pod installed on a cantilever beam. The platform is carried by two operators. Figure S2: learning curves of the SVM, MLP, and the RF used for the superpixel classification. Figure S3: heat map of the absolute value of Pearson's correlation coefficient between all the features.
