# Supplementary material for: Wheat Ear Segmentation Based on a Multisensor System and Superpixel Classification
Source: Plant Phenomics. 2022 Jan 28;2022:9841985. doi: 10.34133/2022/9841985 (PMC8817947; doi:10.34133/2022/9841985)
Supplement: Supplementary Materials — Table S1: description of the 15 vegetation indices and the normalized RGB components used as features for the machine learning algorithms aiming at separating leaves and ears. Figure S1: phenotyping platform with the sensor pod installed on a cantilever beam. The platform is carried by two operators. Figure S2: learning curves of the SVM, MLP, and the RF used for the superpixel classification. Figure S3: heat map of the absolute value of Pearson's correlation coefficient between all the features. [file 9841985.f1.docx]

Supplementary Materials

| **Index** | **Name** | **Formula** | **Reference** |
| --- | --- | --- | --- |
| NDVI | Normalized Difference Vegetation Index | $\frac{nir-red}{nir+red}$ | (Rouse et al. 1973) |
| GNDVI | Green Normalized Difference Vegetation Index | $\frac{nir-green}{nir+geen}$ | (Gitelson, Kaufman, and Merzlyak 1996) |
| NDRE | Normalized difference Red Edge | $\frac{nir-rededge}{nir+rededge}$ | (Barnes et al. 2000) |
| GRVI | Green ratio vegetation index | $\frac{nir}{green}$ | (Sripada et al. 2006) |
| mNDblue | Modified normalized difference blue | $\frac{blue-red}{blue+nir}$ | (Jay et al. 2017) |
| SR | Simple ratio | $\frac{nir}{rededge}$ |  |
| RDVI | Renormalized difference vegetation index | $NDVI\times(Nir-red)$ | (Roujean and Breon 1995) |
| OSAVI | Optimized Soil-Adjusted Vegetation Index | $\frac{(1+0.16)\times(nir-red)}{nir+red+0.16}$ | (Rondeaux, Steven, and Baret 1996) |
| TDVI | Transformer difference vegetation index | $1.5\frac{nir-red}{\sqrt{nir+red+0.5}}$ | (Roujean and Breon 1995) |
| MSAVI | Modified Soil-Adjusted Vegetation Index | $\frac{(2nir+1-\sqrt{\left( 2nir+1 \right)^{2}-8(nir-red)}}{2}$ | (Qi et al. 1994) |
| MCARI | Modified Chlorophyll Absorption Reflectance Index | $(\left( rededge-red \right)-0.2\times(rededge-green))\times\frac{rededge}{red}$ | (Daughtry et al. 2000) |
| TCARI | Transformed Chlorophyll Absorption Reflectance Index | $3\times((rededge-red)-0.2\times(rededge-green)\times rededge/red)$ | (Haboudane et al. 2002) |
| VARI | Visible Atmospherically Resistant index | $\frac{green-red}{green+red-blue}$ | (Gitelson et al. 2002) |
| CIrede | Red Edge chlorophyll index | $\frac{nir}{rededge}-1$ | (Roujean and Breon 1995) |
| CIgreen | Green chlorophyll index | $\frac{nir}{green}-1$ | (Roujean and Breon 1995) |
| R | Normalized red | $\frac{r}{r+g+b}$ | **/** |
| G | Normalized green | $\frac{g}{r+g+b}$ | **/** |
| B | Normalized blue | $\frac{b}{r+g+b}$ | **/** |

**Table S1**. Description of the 15 vegetation indices and the normalized RGB components used as features for the machine learning algorithms aiming to separate leaves and ears. r, g and b are the channels of the RGB camera. blue, green, red, rededge and nir are the channels of the multi-spectral camera array, respectively at 490, 550, 680, 720 and 800 nm.

**
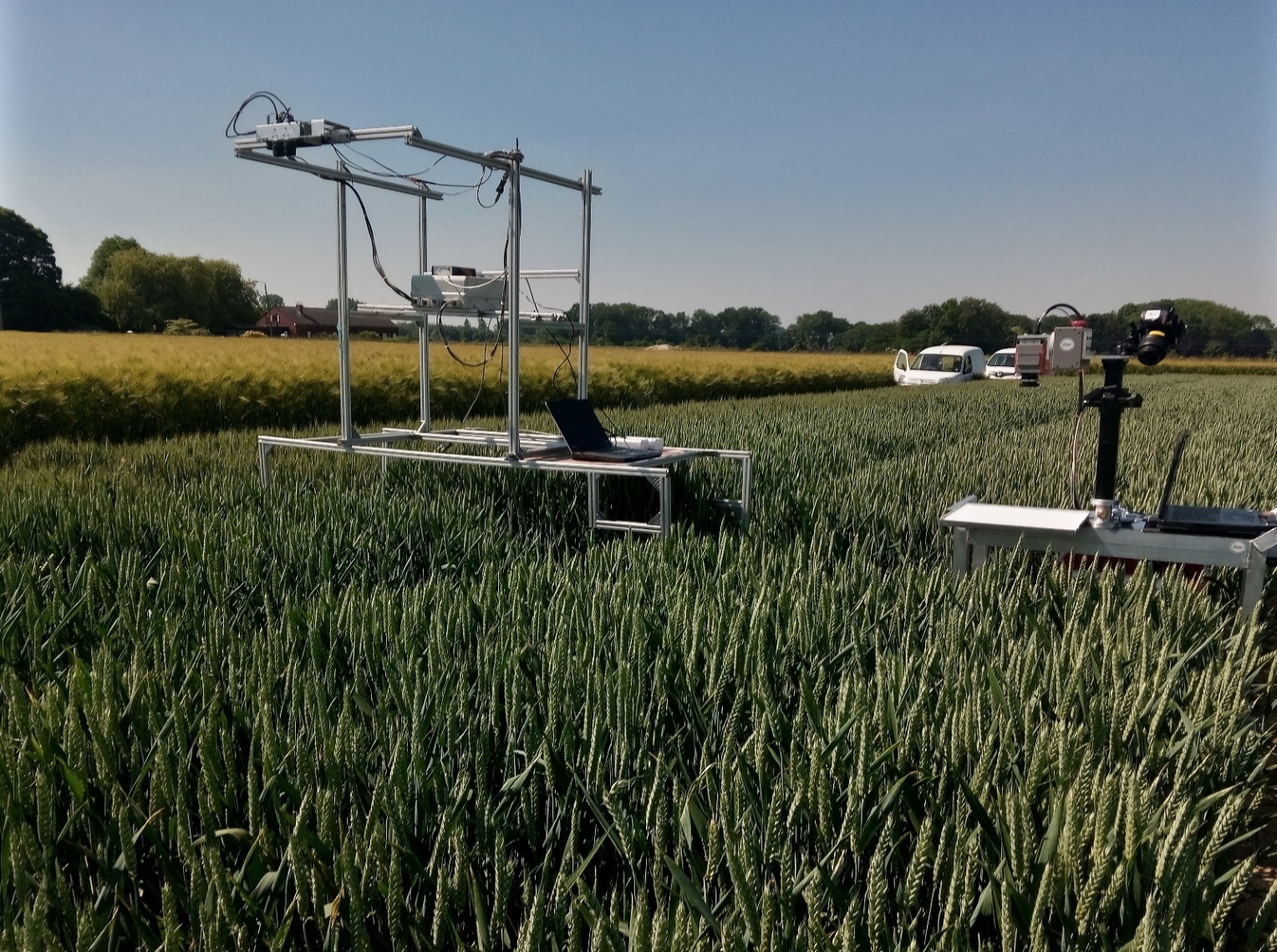
**

**Figure S1.** Phenotyping platform with the sensor pod installed on a cantilever beam. The platform is carried by two operators.

**
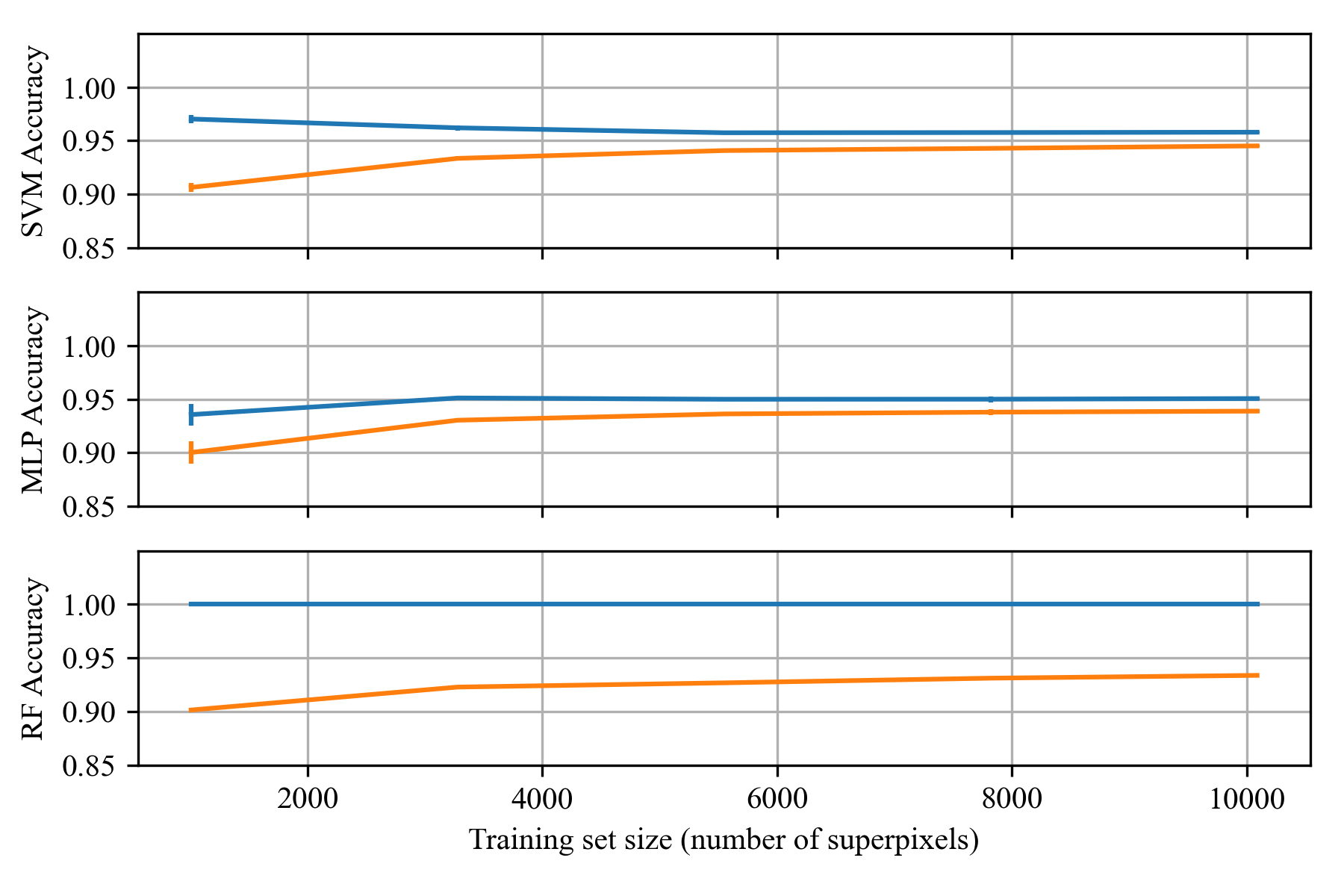
**

**Figure S2.** Learning curves of the SVM, MLP and the RF used for the superpixel classification. Blue line refers to the average train score and the orange line refers to the average test score from a 5-fold cross-validation on the training set.


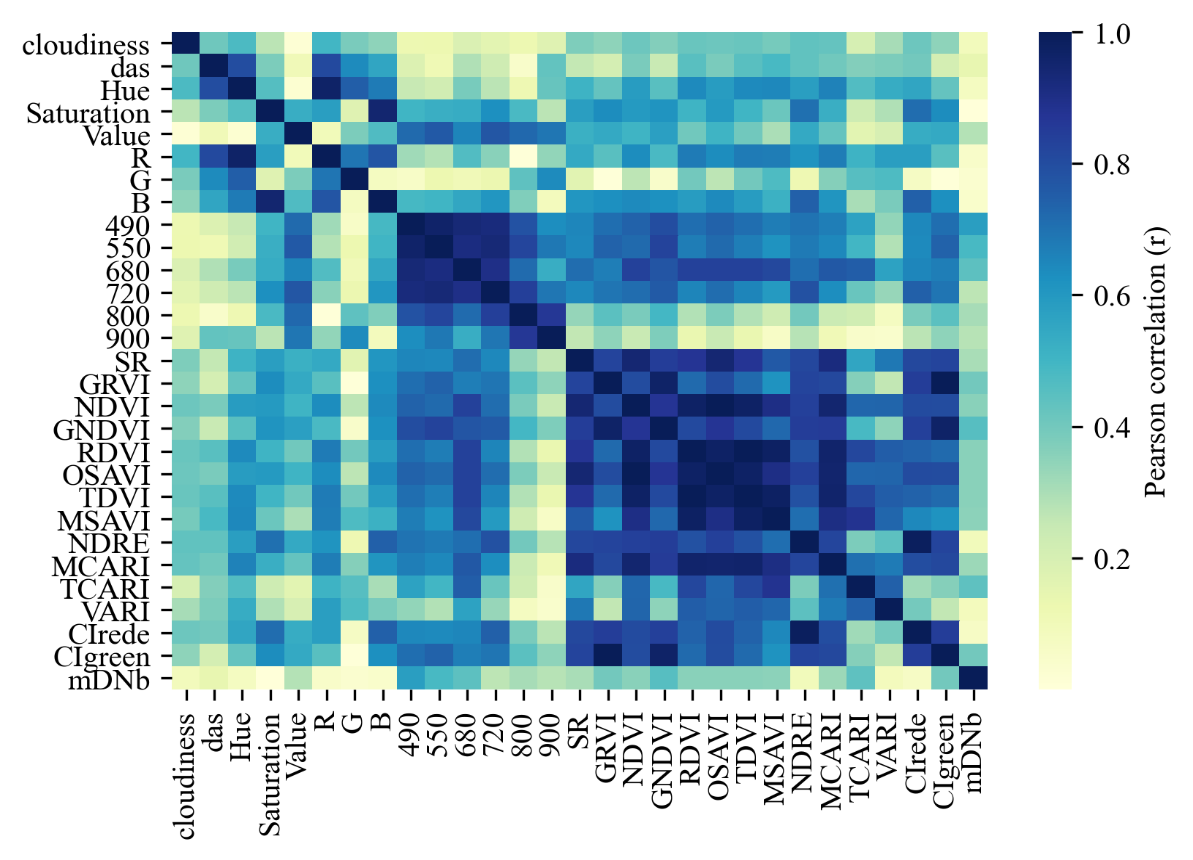


**Figure S3,** Heat map of the absolute value of Pearson’s correlation coefficient between all the features

Barnes, Em M, TR R Clarke, Se E Richards, PD D Colaizzi, J Haberland, M Kostrzewski, P Waller, et al. 2000. “Coincident Detection of Crop Water Stress, Nitrogen Status and Canopy Density Using Ground Based Multispectral Data.” In *Proc. 5th Int. Conf. Precis Agric*.

Daughtry, C. S.T., C. L. Walthall, M. S. Kim, E. Brown De Colstoun, and J. E. McMurtrey. 2000. “Estimating Corn Leaf Chlorophyll Concentration from Leaf and Canopy Reflectance.” *Remote Sensing of Environment* 74 (2): 229–39. https://doi.org/10.1016/S0034-4257(00)00113-9.

Gitelson, Anatoly A., Yoram J. Kaufman, and Mark N. Merzlyak. 1996. “Use of a Green Channel in Remote Sensing of Global Vegetation from EOS- MODIS.” *Remote Sensing of Environment* 58 (3): 289–98. https://doi.org/10.1016/S0034-4257(96)00072-7.

Gitelson, Anatoly A., Yoram J. Kaufman, Robert Stark, and Don Rundquist. 2002. “Novel Algorithms for Remote Estimation of Vegetation Fraction.” *Remote Sensing of Environment* 80 (1): 76–87. https://doi.org/10.1016/S0034-4257(01)00289-9.

Haboudane, Driss, John R. Miller, Nicolas Tremblay, Pablo J. Zarco-Tejada, and Louise Dextraze. 2002. “Integrated Narrow-Band Vegetation Indices for Prediction of Crop Chlorophyll Content for Application to Precision Agriculture.” *Remote Sensing of Environment* 81 (2–3): 416–26. https://doi.org/10.1016/S0034-4257(02)00018-4.

Jay, Sylvain, Nathalie Gorretta, Julien Morel, Fabienne Maupas, Ryad Bendoula, Gilles Rabatel, Dan Dutartre, Alexis Comar, and Frédéric Baret. 2017. “Estimating Leaf Chlorophyll Content in Sugar Beet Canopies Using Millimeter- to Centimeter-Scale Reflectance Imagery.” *Remote Sensing of Environment* 198 (September): 173–86. https://doi.org/10.1016/j.rse.2017.06.008.

Qi, J., A. Chehbouni, A. R. Huete, Y. H. Kerr, and S. Sorooshian. 1994. “A Modified Soil Adjusted Vegetation Index.” *Remote Sensing of Environment* 48 (2): 119–26. https://doi.org/10.1016/0034-4257(94)90134-1.

Rondeaux, Geneviève, Michael Steven, and Frédéric Baret. 1996. “Optimization of Soil-Adjusted Vegetation Indices.” *Remote Sensing of Environment* 55 (2): 95–107. https://doi.org/10.1016/0034-4257(95)00186-7.

Roujean, Jean Louis, and François Marie Breon. 1995. “Estimating PAR Absorbed by Vegetation from Bidirectional Reflectance Measurements.” *Remote Sensing of Environment* 51 (3): 375–84. https://doi.org/10.1016/0034-4257(94)00114-3.

Rouse, J. W., R. H. Hass, J.A. Schell, and D.W. Deering. 1973. “Monitoring Vegetation Systems in the Great Plains with ERTS.” *Third Earth Resources Technology Satellite (ERTS) Symposium* 1: 309–17. https://doi.org/citeulike-article-id:12009708.

Sripada, Ravi P., Ronnie W. Heiniger, Jeffrey G. White, and Alan D. Meijer. 2006. “Aerial Color Infrared Photography for Determining Early In-Season Nitrogen Requirements in Corn.” *Agronomy Journal* 98 (4): 968–77. https://doi.org/10.2134/agronj2005.0200.
